# Whole-brain imaging reaches new heights (and lengths)

**DOI:** 10.7554/eLife.13367

**Published:** 2016-01-20

**Authors:** Alexandre Albanese, Kwanghun Chung

**Affiliations:** 1 Institute for Medical Engineering and Science, Massachusetts Institute of Technology, Cambridge, United States; 2 The Picower Institute for Learning and Memory, Massachusetts Institute of Technology, Cambridge, United Stateskhchung@mit.edu; 3 The Institute for Medical Engineering and Science, Massachusetts Institute of Technology, Cambridge, United States; 4 The Department of Chemical Engineering, Massachusetts Institute of Technology, Cambridge, United States

**Keywords:** Neuroanatomy, Whole-brain imaging, Axonal reconstruction, Tissue clearing, Neuroinformatics, Neuroimaging, Mouse

## Abstract

Advances in microscopy and sample preparation have led to the first ever mapping of individual neurons in the whole mouse brain.

**Related research article** Economo MN, Clack NC, Lavis LD, Gerfen CR, Svoboda K, Myers G, Chandrashekar J. 2016. A platform for brain-wide imaging and reconstruction of individual neurons. *eLife* **5**:e10566.doi: 10.7554/eLife.10566

**Image** A population of sparsely labeled neurons in a mouse brain

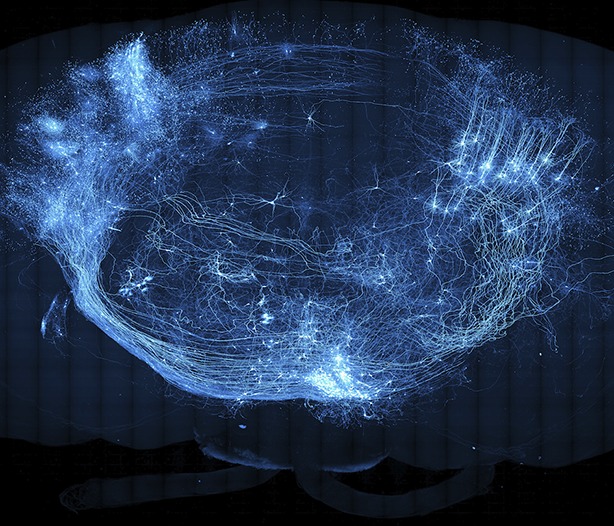

For decades, neuroscientists have dreamed of being able to produce a connectome – a map that shows how the neurons in the mammalian brain are connected together. However, individual neurons are rather small, so we need a microscope to see them with enough detail to produce a connectome. But how do you image a whole brain with a microscope? The answer is: you slice it!

The first connectome was produced in the 1980s and showed the 302 neurons in *C. elegans,* a small round worm (White et al., 1986). This feat required sectioning the worm into slices that were just 50 nm (50 x10^−9^ meters) thick, imaging them using electron microscopy, and then manually aligning thousands of printed micrographs to reconstruct each neuron down to its finest details. Modern versions of this technique have been used to produce connectomes for the mouse retina (Kim et al., 2014) and for small pieces of the mouse cortex (Kasthuri et al., 2015; Bock et al., 2011; Figure 1a).

**Figure 1.**
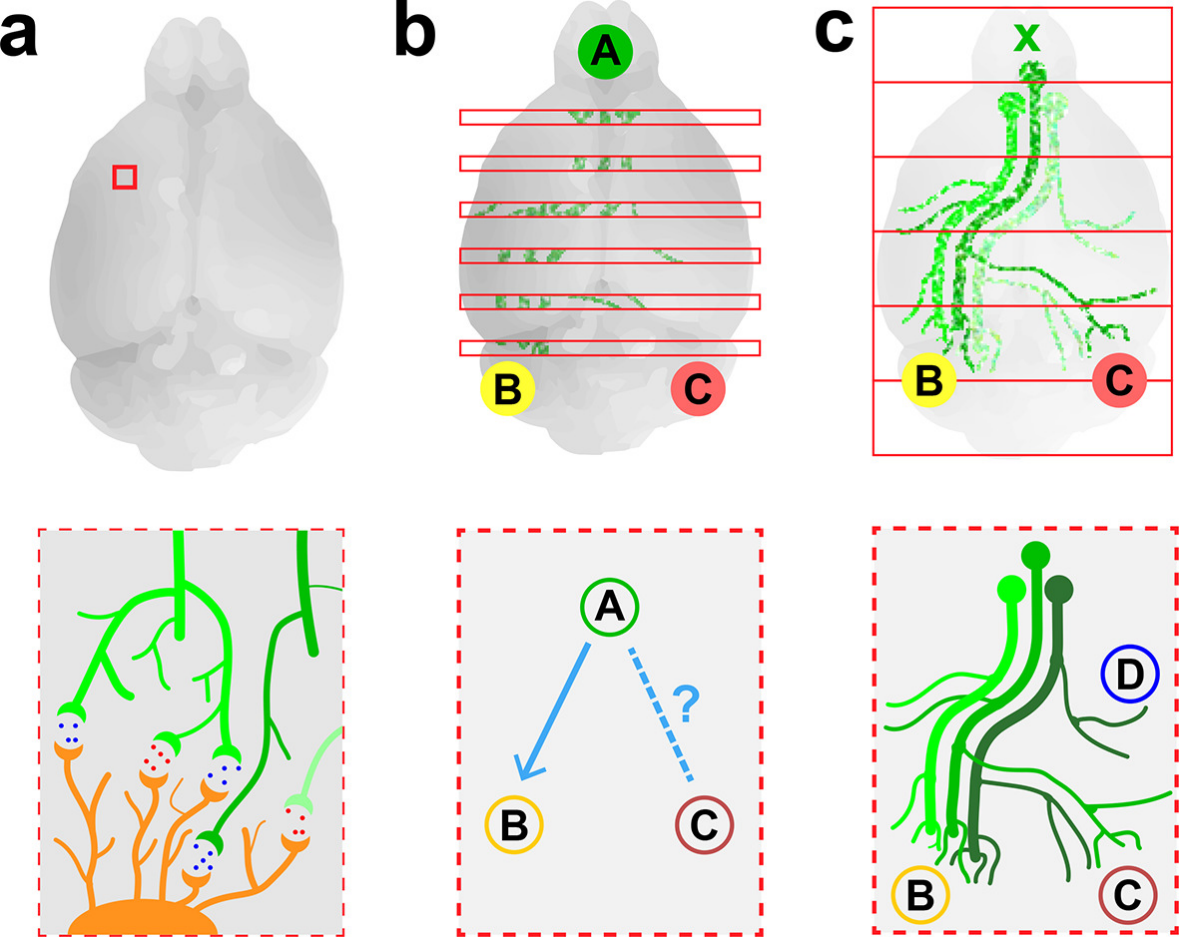
Imaging neurons in the brain. (****a****) Electron microscopy can be used to map the neurons in a small volume of brain tissue (typically about 0.015 mm^3^; top) by recording images of thousands of very thin slices and combining them. This approach can provide a high-resolution connectome of a small volume of tissue (bottom). (**b**) Fluorescence microscopy can be used to map the connections between the different regions of a brain by recording images of about 150–300 slices separated by about 0.05–0.1 mm (top) and combining them. The resulting projectome can reveal, for example, that region A is connected to region B, but not to region C (bottom). (**c**) Economo et al. used a combination of tissue clearing, serial sectioning and sparse labeling (by injecting adenovirus at x; top) to track the projections from 10–50 neurons throughout the brain (bottom). This approach allowed projections with diameters that measured as little as 100 nm to be mapped. This technique provides single-neuron mapping throughout the whole brain. Illustrations are not to scale.

Electron microscopy is an effective method for visualizing the highly connected networks formed by neurons in relatively small samples, such as a worm or a small piece of tissue, but it is too slow to be used on larger samples. Therefore, in order to produce projectomes – maps that show how the different regions of the mammalian brain are connected to each other – neuroscientists have turned to a form of microscopy called fluorescence microscopy. This technique, which sacrifices resolution in favor of speed, can scan large areas and provides sufficient resolution to map the connections between different regions in large samples of brain tissue.

Researchers at the Allen Institute for Brain Science recently used fluorescence microscopy to produce the first brain-wide projectome for the mouse brain (Oh et al., 2014). This involved using automated vibratomes and two-photon microscopes to slice and image brains at intervals of 0.1 mm (Ragan et al., 2012). However, the use of such a large interval meant that it was not possible to track individual neurons (Figure 1b).

Now, in *eLife*, Jayaram Chandrashekar of the Janelia Research Campus and colleagues – including Michael Economo and Nathan Clack as joint first authors – report that they have used fluorescence microscopy to visualize whole neurons in a mouse brain (Economo et al., 2016). To make this breakthrough Economo et al. combined a technique called tissue clearing with automated tissue sectioning and imaging to obtain a three-dimensional image of the entire brain (Figure 1c). Tissue clearing is a sample preparation strategy that renders a biological tissue optically clear by removing lipids and/or introducing a medium that has the same refractive index as the cells. Economo et al. tested a number of emerging tissue clearing techniques – such as CUBIC (Susaki et al., 2015), CLARITY (Chung et al., 2013) and iDISCO (Renier et al., 2014) – but none of them were compatible with tissue sectioning, so they developed a partial tissue clearing protocol that allowed fluorescence from up to 0.25 mm below the surface of the tissue to be detected.

Brains were imaged for about 10 days with a custom-built high-speed two-photon microscope, generating 10 TB of data. To track the projections from individual neurons over long distances (and through large numbers of other neurons), Economo et al. used a technique called sparse labeling: this involved injecting a low dose of adenovirus into the brain so that only 10–50 neurons were labeled. The projections from these neurons could then be mapped throughout the brain by recording fluorescence from the adenovirus (to which fluorescent labels had been attached). Manual reconstructions revealed that just five neurons innervated some 28 different regions of the brain and covered distances over 300 mm.

The combination of tissue clearing and serial-sectioning has provided the first ever tracking of individual neurons in the whole brain using optical microscopy. However, this achievement comes with some problems that are familiar when we try to balance speed and high resolution. Continuous whole-brain imaging enables a 10-fold increase in imaging resolution, but takes 10-times longer than the serial imaging studies with intervals of 0.1 mm performed at the Allen Institute. This limits the experimental throughput, the statistical power of observations, and potential applications. And since Economo et al. were only able to image a sparse distribution of fluorescent neurons, producing a whole-brain projectome at single-cell resolution is still out of reach at this point.

Future studies will need to build on this progress made by Economo et al., and may require the development of new technologies that do not require sparse labeling in order to accurately and simultaneously reconstruct many neurons within a single brain. Nevertheless, by bringing neuroscientists closer to the possibility of creating full-resolution projectome maps of the brain, this work represents an important milestone in our understanding of the brain.
